# Health of Refugees and Migrants—Where Do We Stand and What Directions Should We Take?

**DOI:** 10.3390/ijerph16081319

**Published:** 2019-04-12

**Authors:** Osnat Keidar, David S. Srivastava, Emmanouil Pikoulis, Aristomenis K. Exadaktylos

**Affiliations:** 1 Department and Emergency Medicine, Inselspital, Bern University Hospital, University of Bern, 3010 Bern, Switzerland; DavidShiva.Srivastava@insel.ch (D.S.S.); Aristomenis.Exadaktylos@insel.ch (A.K.E.); 2 3rd Department of Surgery, “Attikon” University General Hospital, National and Kapodistrian, University of Athens (NKUA), 11527 Athens, Greece; mpikoul@med.uoa.gr

## 1. Introduction

International migration, particularly to Europe, has increased in the last few decades, making research on aspects of this phenomenon, including numbers, challenges, and successes, particularly vital. Accordingly, we are pleased to introduce a Special Issue of *International Journal of Environmental Research and Public Health* on the health of refugees, migrants, and ethnic minorities. 

Discussions of a topic must begin with foundational definitions: 

A “migrant” is any individual who moves across international borders away from 

his or her country of origin, regardless of legal status or cause [1]. 

A “refugee**”** is any person who, resulting from a well-founded fear of persecution for reasons of race, religion, nationality, membership of a particular social group or political opinion, is outside the country of his/her nationality and is either unable or too scared to avail himself/herself of the protection of that country [2]. 

An “asylum seeker” (AS) is someone who has applied for protection as a refugee and is awaiting the determination of his or her status [3]. The above definitions may vary according to country and local law; however, there remain fundamental distinctions between a migrant and a refugee. In comparison with migrants, refugees have not chosen to leave their country but have fled in response to a crisis. They are more likely to leave family behind, travel without proper documents, have little choice on their country of arrival, and will probably never return home [4]. These unique characteristic impact how migrants and refugees should be considered in terms of both needs and health outcomes. 

Sustainable Development Goals (STGs) aim to decrease disparities within populations by 2030. For example, SDG 10 incorporates Target 10.7: “Facilitate orderly, safe, regular and responsible migration and mobility of people, including through the implementation of planned and well-managed migration policies”; this is intended to guide state members in taking measurable steps to attain these goals [5].

Having provided necessary definitions, some relevant data on migrants, refugees, and asylum seekers includes the following: As of 2017, the estimated number of international migrants has reached 258 million, in comparison with 220 million in 2010 and 173 million in 2000 [6]. As of 2017, Europe and Asia together host 62% of total international migrants [6].

At the end of 2016, the total number of refugees and AS in the world was estimated at 25.9 million, which corresponds to 10.1% of all international migrants. Turkey recorded the largest refugee population and hosts approximately 3.1 million refugees and AS, with the most significant increase in the world since 2000 [6].

During 2015 and 2016, more than 2.5 million people applied for asylum in the European Union (EU). To put such numbers in context, more than 2030 people are thought to have lost their lives in the Mediterranean during the first six months of 2017. In 2015 and 2016, more than 2.3 million illegal crossings were detected by Frontex, the EU border surveillance agency. Within Europe, Germany hosts the greatest number of refugees: approximately 1.2 million, including 222,560 in-process AS requests [7].

The Dublin Regulation establishes the responsibility of a member state to examine the asylum application; the Regulation’s objective is to ensure rapid access to asylum procedures and to guarantee that the merits of the application are examined by a single, clearly determined member state. Criteria for establishing responsibility run, in hierarchical order, from family considerations, to recent possession of a visa or residence permit in a member state, to whether the applicant has entered the EU regularly or irregularly [8].

These figures may spark humanitarian, security, and ethical concerns and may oblige European countries to support the absorption of these refugees. Making the outmost effort to support their new life is not only a humanitarian issue but also an essential obligation of the European countries for both economic and practical reasons. Most of these refugees will, in some years, become migrants and later residents. An effective supportive process will enable them to become an integral and contributing work force in their host countries. However, if this process fails, these new residents may pose a significant economic and security burden on society, as can already be observed in some countries. Unemployment, health disparities, mental problems, and addictions are only some of the outcomes of the failure to create a supportive and effective process for AS, migrants, and refugees. Recent studies indicate that countries with a higher integration score for migrants present better socio-economic and health outcomes for these communities. Therefore, research on these phenomena is essential, including the presentation of needs and assets of these diverse populations as well as suggestions for possible policies, interventions, and subsequent evaluation of these programs.

This Special Issue highlights this necessary and relevant area of research. The Special Issue is not intended solely for academic purposes. Policy makers may use the suggested policies and interventions to improve existing programs.

## 2. Review of Articles in the Special Issue

There are 37 articles in the current special issue, including studies on diverse topics relating to the health of refugees, migrants, and ethnic minorities.

Most articles (28 of them) present studies focusing on European host countries, including Germany, Greece, Italy, Switzerland, Spain, Portugal, and Sweden. The focus on Europe is justified if we take into consideration the increase in numbers of refugees and migrants who have come to Europe in recent years. However, there are also articles which present studies from countries in other continents.

Topics discussed in the Issue’s articles are summarized in Table 1, and include healthcare (HC) utilization, infectious diseases, mother and child health, mental health, and chronic diseases.

### 2.1. Infectious Diseases

Infectious diseases are a frequent topic in this Special Issue, which features six systematic reviews and one commentary, all written as part of ECDC publications. These reviews discuss the effectiveness and cost effectiveness of screening for HIV, hepatitis B and C, schistosomiasis, and strongyloidiasis. The articles also discuss the effectiveness of interventions (including vaccinations) and their cost effectiveness in migrants in the EU/EEA (Table 1) [9,10,11,12,15,16]. These have led to a recent publication of the European Center for Disease Prevention and Control (ECDC) entitled “Public health guidance on screening and vaccination for infectious diseases in newly arrived migrants within the EU/European Economic Area (EEA)” [46].

### 2.2. Mental Health

Mental health is also an important topic and is a major concern within migrant and refugee populations (Table 1). Prevention and treatment are essential. High rates of mental health issues are likely impacted by the trauma experienced during crises, travel time to the host country and the many different challenges related to migrant experiences, including separation from family, difficulties in proper use of the health services as a result of cultural differences, lack of knowledge of the new health system, and language barriers [20,21,22,23,24,25].

### 2.3. Health Care Utilization

This issue also documents how migrants and refugees access health services and some of their health care outcomes [26,27,28,29,30,31,32,33,34,35]. Studies in this special issue emphasize the need for well-structured policies and guidelines in the EU to ensure the proper integration of AS and refugees within health systems. Studies indicate that there are differences in health service consumption among migrant communities in comparison with the host population of a country, with more frequent outpatient discharges, walk-in visits, visits for less urgent reasons, and an increased need for access to psychiatric consultation among migrant populations (Table 1).

### 2.4. Mother and Child Health

Within the refugee and migrant population, children and women are particularly vulnerable. The increase in health service consumption for women poses great challenges and is significantly impacted by differences in culture diversity. This topic requires professional and targeted adaptation of health services if such services are to properly face this challenge and overcome barriers to both appropriate use and better service. Women and girls, as well as health care providers, must be instructed on these issues (Table 1) [36,37,38,39,40,41,42].

### 2.5. Lifestyle and Chronic Diseases

Chronic diseases and lifestyles are briefly presented in this issue in three articles. As seen in Table 1, migrant status does not necessarily pose a risk factor. Other sociodemographic factors, such as gender, age, education, and religious adherence were found to be associated with obesity and overall quality of diet, such as consumption of vegetables and fruit as well as alcohol consumption [43,44,45].

## 3. Discussion

The numerous articles in this Special Issue illustrate the increased interest and new data on the unique needs of migrant and refugee populations. Migrant and refugee status poses increased risk and health challenges; host countries are encouraged to integrate policies and interventions to accommodate these gaps.

The Ottawa charter and the social determinants of health are linked to migration. Within the total migrant population, refugees and asylum seekers are at greater risk of poor health outcomes. Most refugees come from low income countries, with a high burden of both disease and civil unrest. They suffer from unfortunate conditions during their transit and, occasionally, following their arrival in the host country. Other challenges include legal status, sometimes lack of culturally competent health and social services, lack of social support and isolation, and difficult working and living conditions [47,48,49].

Other factors that impact migrant and refugee health include social and cultural barriers to integration, stress, exclusion and discrimination, poor socioeconomic status, loss of supportive networks, and changes in lifestyle and diet. This has encouraged the WHO and other organizations to include migration as a social determinant of health and to promote inter-sectorial HP initiatives to address these determinants [47,50,51].

A holistic approach is required for interventions that aim to improve migrant health. Such an approach must be inclusive and take into consideration the beliefs, values, capacities, needs, and social context of all migrants and refugees. It also must support integration by using participatory approaches [47,52,53] and be adopted by decision makers [54,55]. Areas for interventions should include the five action areas of the Ottawa charter [56]:

- Ensuring that there are policies within all sectors of government which aim to promote the health of refugees and migrants;
- Improvements in social services, and the quality of physical and social environments, prioritizing community-centered approaches that build local capacities;
- Investment in language support and health literacy initiatives to develop personal skills;
- Promotion of approaches to health care that are sensitive to culture and diversity;
- Development of a culturally competent health workforce [47,57].

However, additional research is required, as discussed in both the articles of the Special Issue and elsewhere [55,58]. More studies are needed that use both qualitative and quantitative methods to enhance our understanding of the current situation and the various factors which impact migrant and refugee health. Many studies focus on specific countries or populations, which implies that their conclusions may not be applicable to global migrant communities. As evaluation of HP interventions has been inadequate, both process and impact evaluation should be incorporated in all programs. This is to assess the effectiveness of such initiatives, and to support the improvement and focus for sub-groups from various backgrounds, languages, and cultures.

It is our ethical responsibility, as both providers and policy makers, to integrate these approaches and focuses in our daily work to help to support the health gaps and to promote equity.

## 4. Conclusions

Migrant and refugee health poses a significant challenge. Further development of guidelines and policies at both local and international levels is needed. Priorities must be set by encouraging and funding in-depth research that aims to evaluate the impact of existing policies and interventions. Such research will help us formulate recommendations for the development of strategies and approaches that improve and strengthen the integration of migrants and refugees into the host countries.

## Figures and Tables

**Table 1:** Summary of special issue manuscripts, arranged according to principle topics.

Topic	Main Findings	Number of Articles
Infectious diseases	Early detection of infections is important to prevent morbidity and mortality. The most important issues include the effectiveness and cost effectiveness of screening for HIV, hepatitis B and C, schistosomiasis and strongyloidiasis [9,10,11,12,13], Older people are at greater risk of developing active tuberculosis, so that new and better policies and strategies are needed for the detection and treatment of tuberculosis in older people [14] In migrant centers, the staff numbers and their various skills, together with physical infrastructure (including poor hygiene, lack of electricity and heating) are crucial in preventing and mitigating outbreaks [13]. A review on interventions to increase vaccine intake found that they had little impact on vaccine uptake [15]. Factors associated with accessibility and acceptability of interventions to prevent infectious disease included knowledge of the disease and the related stigma, as well as migrants’ interaction with HC providers [16]. HC providers must possess culture-sensitive communication skills when planning interventions [16]. They must focus on social mobilization and community outreach when planning vaccination programs and educational campaigns [15].	11 [9,10,11,12,13,14,15,16,17,18,19]
Mental health	Migrants face traumatic events that impact their mental health, with various risk factors, including association between early trauma and depression symptoms and feelings of personal failure [20]. In comparison to controls, North Korean refugees with suicide ideation had lower levels of family cohesion, lower self-esteem, lower resilience and higher post-traumatic stress disorder [21]. AS with rejected applications showed high levels of psychiatric emergencies and higher stress levels [22]. In women with HIV infection, mental health was impaired by multiple factors, including stigma, racial discrimination and resettlement adversities [23].	6 [20,21,22,23,24,25]
Healthcare utilization	Primary care is most often accessed in the first period directly after arrival [26]. In comparison with the local population, migrants (mainly from North Africa) use ER more often and for less urgent complaints and are more often discharged as outpatients; young males more often consulted psychiatrists [27]. Migrants are more often involved in hospital-related aggression [28]. The health insurance card increased the use of outpatient care [29]. It is important to develop guidelines, policy and resources to support the health system; NGOs’ support is essential for successful integration [30].	10 [26,27,28,29,30,31,32,33,34,35]
Mother and child health	An increased need for reproductive health services. Barriers faced by women to access services include language, stigma, direct and indirect cost, lack of cultural competency within health services, distance and difficulty in navigating health services [36,37]. Healthcare providers emphasize the unique needs of migrant and refugee women in sexual and reproductive health, and the lack of proper training to address them [38]. Sexual violence is highly frequent in migrants and refugees in Europe [39]. Displaced or migrant girls and young women in Africa possessed limited knowledge of contraceptive methods, STIs and HIV/AIDS. This poses a risk to gender and sex-based violence and abuse [40]. Need to train healthcare providers’, in order to increase culturally-tailored health services [36,38].	7 [36,37,38,39,40,41,42]
Lifestyle and chronic diseases	Being a migrant woman was a risk factor for obesity, while migrant men of low educational level in the Spanish population were relatively protected [43] Older and educated women consumed more vegetables and fruits than men [44] Intrinsic religiosity is a protective factor for smoking and alcohol consumption in Polish migrants in Germany [45]	3 [43,44,45]
